# Motivations to pursue careers in health research: A qualitative study of underrepresented faculty in the health sciences

**DOI:** 10.1017/cts.2025.10178

**Published:** 2025-11-03

**Authors:** Nancy Gauvin, Holly Thomas, Hemika Vempalli, Gretchen E. White, Natalia E. Morone, Audrey J. Murrell, Megan E. Hamm, Marie K. Norman, Doris Rubio

**Affiliations:** 1 School of Health and Rehabilitation Sciences, University of Pittsburgh, Pittsburgh, PA, USA; 2 Department of Medicine, University of Pittsburgh, Pittsburgh, PA, USA; 3 Institute for Clinical Research Education, University of Pittsburgh, Pittsburgh, PA, USA; 4 Chobanian and Avedisian School of Medicine, Boston University, Boston, MA, USA; 5 School of Business, University of Pittsburgh, Pittsburgh, PA, USA

**Keywords:** career motivations, qualitative study, early career investigators, underrepresented background, workforce

## Abstract

**Introduction:**

To successfully recruit and retain faculty members from underrepresented backgrounds (URBs), we need to understand the factors that attract them to research careers in the first place. However, scholarship in this area has focused largely on students who are contemplating research careers rather than faculty members who are currently *in* such careers.

**Methods:**

This study explores the career motivations of early-career health researchers (faculty members and postdoctoral fellows) from URBs. It was conducted as part of a cluster randomized trial across 25 academic institutions investigating a support intervention. We conducted 1-hour semi-structured qualitative interviews with scholars from URBs in both the intervention and control arms of the trial. To our knowledge, this is the largest qualitative study of early-career faculty members from URBs to date.

**Results:**

Seventy-eight individuals were interviewed. Our analysis revealed six key themes pertinent to participants’ motivations to pursue research careers: (1) love of science; (2) making a larger impact; (3) happenstance and economic considerations; (4) family, community, and a path out of poverty; (5) the role of mentors and role models; and (6) support programs for scholars from URBs.

**Conclusions:**

Our results align with prior studies while offering new insights into the motivations of URB faculty members in research careers. These insights can and should inform the design of programs to both recruit and retain URM faculty members in research careers.

## Introduction

Diversity in the scientific workforce is essential for conducting high-quality, transformative science. Research shows that workforce diversity is linked to improved problem-solving [1], increased innovation [2–6], and greater academic productivity [7,8]. In the health sciences more specifically, diversity in the research workforce has proven critical for addressing health inequities and creating greater impact through community-engaged approaches [9–11]. Given the importance of bringing a range of backgrounds, experiences, and ideas to bear on healthcare problems, it is critical to understand *factors that motivate or inhibit individuals from varied backgrounds to pursue research careers* in the health sciences.

Ample scholarship has investigated barriers to attracting and sustaining scholars from diverse backgrounds in academic and research careers [12–15]. These barriers include, but not limited to, lack of exposure to research at early ages [16], financial barriers [17], imposter syndrome [18], funding disparities [19], academic racism [20–23], and an unacknowledged “hidden curriculum” [24] that exacerbates inequities). However there is limited scholarship on the motivations that drive scholars from diverse backgrounds to begin with [25–27], and it tends to focus either on the impact of specific training programs or on the motivations of high school and undergraduate students [25–27]. Data on students are helpful to a point, but they do not tell the whole story as later life events can influence career paths profoundly and motivations change over time [28]. There is a need for research that explores the perspectives and motivations of researchers who are currently engaged in research careers, not simply contemplating them.

In this study, we focus on the career motivations of early-career faculty members and postdoctoral fellows in research careers, using qualitative interviews and content analysis. We used a qualitative approach to explore how URB individuals understand their motivations to pursue research careers, framed in richer context than survey data allow.

## Methods

Building Up was a cluster-randomized trial conducted at 25 academic institutions from 2020–2021, comparing two 10-month-long interventions seeking to support the careers of postdoctoral fellows and early-career faculty members from URBs. Participants in the intervention arm were assigned to a near-peer (mid-career) mentor at their institution and offered networking and coursework in manuscript and grant writing. In the control arm, participants received the usual mentoring, networking, and coursework offered by their institutions. Both groups attended a monthly leadership webinar. Study participants completed up to 4 annual surveys (pre-intervention, post-intervention, 1-year post-intervention, and 2-years post-intervention). Details of Building Up have been previously described [29,30].

### Recruitment and sampling

The study team recruited site champions to recruit participants. Inclusion criteria included the following: participants had to be from a URB as defined by NIH criteria [36], hold a terminal degree, be a postdoctoral fellow or early-career faculty member, be committed to a research career, and have at least 50% protected research time. Two-hundred and twenty-four postdoctoral fellows and early-career faculty members from URBs participated in the study. To ensure that participants from each institution were interviewed, the study statistician used stratified randomization (by institution) with block sizes of 2 to identify participants that should be approached for interviews.

### Data collection

We collected demographic information (birth year, gender, race/ethnicity, degree type, career stage, disability, educational level of adult who raised them) via survey prior to the intervention. We conducted one-hour, semi-structured interviews one year after the intervention concluded, including scholars from both arms of the trial.

Interview questions focused on participants’ experiences with the programmatic elements of the intervention. However, several other questions were asked about career motivations, challenges, and accomplishments. The present analysis focuses on participants’ responses to the question, “What motivated you to pursue a research career?” Interviews were audio-recorded and transcribed verbatim with names, institutions, and other identifying information omitted.

### Data analysis

78 individuals participated in interviews. Interview audio was transcribed verbatim. One member of the research team (MN) conducted open coding and developed a codebook in MaxQDA, a qualitative data analysis software. A second team member (NG) then reviewed and revised the codebook. MN and NG co-coded 50% of the transcripts and adjudicated coding discrepancies to full consensus. They then conducted content analysis of the coded data, arriving at a set of themes and supporting quotations. MN and NG also met periodically with two other research team members (HT and HV) to discuss codes and themes during the analysis process. Demographic information was matched to question responses to analyze the relationship between subgroups and analytical themes. Analysis was conducted according to the tenets of qualitative description, a theoretical framework developed specifically for health research [31].

## Results

While describing their motivations for pursuing research careers, participants identified a range of challenges they had encountered on their career paths. These challenges included financial struggles, educational hurdles, feelings of isolation in academia, difficulty navigating the “hidden rules” of academia, experiences of racism and bias, cultural and language barriers, and immigration challenges. Many participants reported that limited access to mentorship and professional networks exacerbated these difficulties, making it harder to secure funding and advance their careers. Others highlighted the pressure to balance research responsibilities with personal and familial obligations, which added to the stress of academic life. Despite – and sometimes in explicit defiance of these obstacles – participants expressed deep commitment to their research.

We discuss these challenges in depth in forthcoming publications but mention them here briefly to provide context and highlight the importance of understanding the motivations of early-career researchers from URBs, as they pursue research careers *despite* such challenges.

Our data reflected six principal themes: (1) love of science; (2) making a larger impact; (3) happenstance and economic considerations; (4) family, community, and a path out of poverty; (5) the power of mentors; and (6) support from programs for scholars from URBs. These themes are outlined below with representative quotes in Table 1.

**Table 1.** Themes and representative quotes

Theme	Representative quotes
Love of science	When I was exposed to research experimental projects during medical school, that is where I found myself. **I really was very excited [and have] memories about me being challenged with, you know, thinking out of the box and trying to resolve problems and just you know testing hypotheses, and I thought that was fascinating.** (Int 572, Hispanic woman).*I loved it because I really liked* ***how, like, just the puzzle pieces fitting together***. *(Int 612, demographic information not provided)* **That whole process of life in front of you growing and putting all that information together and analyzing and seeing how it can go**. Well, it was fascinating. (Int 572)
Making a bigger impact	*So, I was actually in pharmacy school like… six years straight through like pharmD program.* ***But about midway through I decided, you know, I really want to have more clinical impact. I, like, really want to help people at a population scale instead of just like you know dispensing medications.*** *(Int 429, Hispanic man)* *And so* **, *I basically just extended my work out in the community implementing and evaluating programs, particularly among under-resourced Spanish-speakers, and then that’s when I decided, okay, well I really like working out in the community, I really like doing this aspect of research.*** *(Int 403, Hispanic woman)* *I realized, I mean working as a clinician there’s so far, or the impact that I can make – it’s only to those who I see in the clinic, right? But* ***I realized that most of these conditions that come into clinic are preventable.*** *I had several research questions that there were no answers to.* ***So, I decided to get this [master’s degree] so I’m able to ask my own research questions and carry out analysis, run my get my results, interprets my result and be able to inform and public health decisions so that way my work goes beyond the clinic. It affects a broader population.*** *(Int 429, Hispanic man)*
Chance and happenstance	*As far as a career as a scientist, my parents are in science. My mother taught science for 40 plus years, and my father worked as a chemist in a paint company. So, I grew up always being exposed to science.* ***But as far as research, I’ve gotten into research kind of a happenstance type thing***. *In college, I heard that you could make a little extra money by becoming a research assistant. But what I did not expect was to really like research. (Int 567, Black woman)* *It’s actually* ***not what I had originally envisioned for myself…*** *When I went to undergrad, I didn‘t know what a PhD was, or [what] research was, and I thought, okay; well, once I finish, you know, college at [University], I’m done … actually there’s a lot more. And so, I was part of the sociology honors program at that time and I was able to work on an undergraduate thesis, and there were about twelve of us in that program. …so that’s how I started getting involved in research. (Int 422, Hispanic woman)* ***When I graduated, to be perfectly honest [laugh] I just didn‘t know what I wanted to do, so I applied to grad school and got in, and went***. *It was more about avoiding making real choices and finding a job, but it turned out to be great for me. I loved doing grad school and I love doing research, so I am still plugging away at it now. (Int 513, American Indian woman)*
Family, community, and a path out of poverty	***I was probably groomed into it, just “cause my parents are both nurse*** *s and I just kinda grew up with them, their influence. (Int 490, Black woman)* *Unfortunately, [my mother] passed the same semester that she was graduating from her master’s in administration. And I took that very personally.* ***I also knew that it was very, it was my only ticket away from poverty*** *… after she passed because she was the breadwinner. We really struggled financially. I [am] the oldest in my family, I have three younger siblings. I just follow the path because it was the only way out of poverty that I knew. (Int 420, Hispanic man)* ***My mother had a lot of mental illness*** *…and she ended up having or developing metastatic lung cancer. And so … I had kind of been interested in the …pathophysiology of cancer, and then, I think, going through that course with her, and just kind of seeing …really both sides of medicine: good and bad. (Int 101, Black woman)*
The role of mentors	*I realized that* ***I really do enjoy doing research and, you know, that’s largely a function of …my mentors*** *challenging me to, like, re-think career goals. (Int 422, Hispanic woman)* *My mentor for one of the summers was [name], who is an adolescent doctor and was really pivotal in me going to med school and really took me under her wing and* ***helped me through the whole process which, I don‘t know if I would be in this position if I hadn’t had her mentorship during college***. *(Int 181, Hispanic man)* *I had* ***really good mentors in [my] fellowship who encouraged me to seek out research positions*** *if available or continue trying to pursue a research career, even if I accepted a clinical position. (Int 286, Black woman)*
Support from programs for URB scholars	***I did a program called the McNair program and I got started in research that way***. *And I ended up presenting at conferences … and I just really loved it. And so, then I ended up you know touring different grad schools with the McNair program. (Int 469, Black woman)* *I think, because I got into that program — and it was a… program pretty much for minority scholars—because I got into that program* **, *I think that research experience allowed me to go to graduate school***. *(Int 369, Black woman)* ***I think I would have really struggled with getting to where I’m at if I hadn‘t had the additional support from that program***, *so from the, like, peer mentorship but also from the, like, people who ran the program, who are all excellent, very supportive, like everybody else in the program. (Int 145, Hispanic woman)*

### Love of science

Most participants described intense intrinsic motivations for pursuing a research career. Indeed, this was the dominant theme that emerged from our data. Participants discussed finding their research fascinating and exciting. The sentiment was consistent throughout many interviews, with one participant describing “falling in love with research.” Several participants noted their love of solving problems. One participant linked his passion for research to the control it afforded him to explore topics of his own choosing. Another participant spoke about the excitement of bringing disparate research interests together in his projects. In several cases, fascination with science began in childhood.

For clinician participants, a love of research was often sparked by clinical experiences and the desire to learn more. In other cases, a passion for research preceded an interest in clinical work and in several cases, as in the following quote.

*[T]here’s a lot of people who go into medicine and then decide, like, okay, I’ll be going into academia within medicine or I’m interested in research.* ***I actually knew that I wanted to do research, even before I knew I wanted to be a clinician. (Int 50, Hispanic woman)***

### Making a larger impact

Participants – both clinicians and non-clinicians – described a strong desire to create meaningful change in health outcomes, though the nature of this motivation varied somewhat by professional background.

Clinician participants often linked their interest in research to perceived limitations of clinical practice. Several expressed frustrations with the inability to address the root causes of their patients’ health issues within the constraints of one-on-one care. For these participants, research offered a means to engage with health problems at a population level, particularly through prevention and systemic change. They viewed research not only as a professional pathway but also as a way to expand their impact beyond the clinical encounter, ultimately serving communities more effectively.

Non-clinician participants, many of whom held PhDs or came from other non-clinical backgrounds, also emphasized a desire to effect large-scale change. However, their motivations were often grounded in commitments to social justice, community engagement, and structural transformation rather than in frustration with clinical limitations. For these individuals, research was seen as a tool for broader impact, rather than a complement or alternative to clinical practice. Their engagement with research was framed less by personal experiences in patient care and more by values-driven goals to contribute to systemic solutions.

Across both groups, participants emphasized a desire to contribute beyond individual success and saw research as a vehicle for societal impact. However, this aspiration was sometimes in tension with traditional academic structures. One participant reflected on the disconnect between research and real-world impact, highlighting concerns about how research is used and disseminated in practice. However, the desire to impact communities was sometimes seen as being in tension with how academic research is used and disseminated, as articulated in this comment from one participant:

*at a certain point, the individuals that we’re studying, they already have cancer, and the research that I’m conducting, it will be published, but the individuals who are mostly impacted by the cancer, they’re not going to read these articles and lots of them don’t even have access to the articles … (Int 622, Black woman)*

### Happenstance and economic considerations

Some participants felt called to science, medicine, and/or research from childhood onward.

For many others, though, the path into research was more a matter of luck than of planning. A number of participants were not initially aware of research careers and found themselves in research by chance (e.g., a summer job led them into research, or a mentor or friend recommended them for a position). In other cases, participants simply found themselves not knowing what to do, took a research-related position, and found that they liked it.

In several cases, financial needs compelled a decision that led to other decisions that eventually led to a career. One participant, for instance, talked about having to take a job at a consulting firm to repay student loans. He found it to be “a very stressful, horrible, difficult place to work,” but the job allowed him to repay his loans, and the company paid for a master’s degree in health and human rights that led him to a research career (Int 674, Middle Eastern man).

### Family, community, and a path out of poverty

Family played a powerful role in participants’ motivations to pursue research careers. Some participants were motivated by the models set by family members who were doctors, nurses, or scientists. In several cases, the experience of family members in science functioned as a cautionary tale, as with one individual who told us:

*Both of my parents were PhD chemists, and they worked for Dow Chemical. Growing up in a small town in Michigan and* ***seeing how the corporation treated people turned me off doing corporate work***. *(Int 556, Black man)*

In several cases, witnessing family members struggle with illness influenced participants’ desire to pursue health careers. As one participant put it: “a family history of diabetes got me interested in diabetes in general.” The same was true for participants who witnessed either kind or callous treatment of loved ones in the healthcare system and who wanted to be clinicians or researchers to improve the healthcare experiences of others, particularly people from marginalized backgrounds. Some participants described growing up poor and cited the desire to escape poverty and find financial stability as one of the reasons they chose their career. For participants who emigrated from other countries, the needs of those countries were often influential in their choice of careers, as well. For example, one person described going into nephrology because there were too few nephrologists in his home country.

### The power of mentorship

Not surprisingly, mentors and teachers played a pivotal role in many of our participants’ career choices. Critical mentorship and guidance came at various life stages and were offered by individuals in roles ranging from high school guidance counselors to undergraduate advisors and professors to faculty mentors, supervisors, and administrators. Mentors provided powerful role models, as in the following case:

*I was…paired with a physician … who was a Black woman and an emergency physician, and she was—she was just, for a lack of a better word, amazing. She was smart and fast and compassionate and forceful, but graceful and beautiful, and she was just; as a Black woman seeing her in action, she was everything I thought I wanted to be, and like, whatever she does, I want to be that. (Int 431, Black woman)*

Participants expressed appreciation for what their mentors taught them, as well as gratitude for their mentors’ generosity in providing access to data sets, authorship on papers, and other professional opportunities, including the opportunity to publish. In several cases, participants credited mentors with keeping them from leaving academia. For instance, one participant described considering applying for industry positions during the COVID-19 pandemic. Her advisor challenged his plans and convinced her to apply for a postdoctoral fellowship instead: “that’s probably the reason why I decided to apply for post-docs, um, rather than just leaving academia.” (Int 422, Hispanic Woman)

In most cases, mentors were a positive influence on participants – but not in all cases. For instance, one participant described a mentor who was “mostly invested in his own growth” and did not provide support (Int 572, Hispanic woman). Another participant described her experience with an ineffective supervisor whose husband was “a textbook bully.” (Int 602, Hispanic transgender woman). Ironically, those negative mentoring experiences led her to one of her primary research interests: advocacy to address bullying and harassment within academia.

### Support from programs for scientists from URBs

Experiences in programs designed to expose undergraduates to research or programs to build knowledge, skills, and connections among scientists from URBs often provided participants with early exposure to research as well as mentorship. Several participants credited these experiences with setting them on their current path. Considerable gratitude was expressed for such programs, exemplified in this quote:

*while I was at the four-year institution, I joined with the minority science programs…* ***I would say that they really were responsible for propelling me into higher education***, *because I had no plans ever to have [a] PhD And even a college degree while I was in high school was, you know, something that I didn’t necessarily plan on having. But* ***they provided the background, the education, the experience really to give me that confidence to apply to graduate school***, *and it was part of the requirement of the program that we apply to graduate school, so I did so.* [Int 452, Hispanic man]

## Discussion

Understanding the motivations of underrepresented researchers in pursuing and persisting in research careers is crucial for improving recruitment and retention strategies. Our study contributes to a growing body of literature that examines these motivations, providing insights that extend beyond high school and undergraduate students to focus on researchers at later stages. Prior research has largely centered students’ initial interest in research careers [25,26], but our findings suggest that motivations are dynamic and influenced by a range of personal, structural, and institutional factors throughout a researcher’s career trajectory.

A central theme was a deep and enduring passion for science and discovery. Participants described an intrinsic love of inquiry, problem-solving, and the generation of new knowledge. This observation expands earlier research on the role of intrinsic motivation in drawing high-school and college students to research careers [25,26] by suggesting that strategies to recruit and retain URB faculty should continue to highlight the satisfactions of scientific discovery throughout a research career [32]. Yet passion alone proved insufficient. Participants consistently emphasized that institutional support was critical, and that targeted programs often transformed initial exposure to research into long-term career commitment.

Many researchers were also motivated by a desire to make a tangible social impact. They expressed a strong commitment to addressing health disparities and scientific gaps that directly affect their own communities and drive social change, echoing findings by Jackson *et al* [10]. Our results extend this insight by demonstrating that the desire to impact communities persists and even deepens as individuals advance into faculty roles, underscoring the need for institutions to value and resource mission-driven, community-engaged research agendas. Mentorship emerged as another decisive factor. Participants credited specific mentors with building confidence, offering strategic career guidance, and helping them navigate the complexities of academia, reinforcing earlier work that highlights the critical role of mentorship in the retention of underrepresented researchers [31–35].

Translating these insights into practice calls for more than broad appeals for institutional change. Our data points to several actionable steps. Universities can implement structured mentorship and sponsorship models that pair early-career investigators with experienced faculty, provide mentor training in equity-centered practices, and formally recognize or compensate effective mentors [31,33,34,35]. Institutions can create bridge or seed-funding mechanisms designed specifically for early-career URB researchers to help them maintain momentum between grants and pilot high-risk, high-impact projects. Equally important for helping investigators chart viable careers in research are clearly articulated promotion and tenure criteria, dedicated research development staff, and accessible workshops on grant writing and institutional resources.

Finally, family and community-responsive policies, such as on-campus childcare and flexible work arrangements, can mitigate the competing responsibilities that often lead to attrition.

These recommendations align closely with what we heard from our participants, many of whom did not grow up affluent, and sought financial stability while simultaneously facing challenges in securing funding and balancing professional and personal demands. Our findings suggest that researchers from URBs persist when they can see a realistic path to stability, when they receive consistent mentorship, and when their institutions actively value the community-impactful science they are driven to pursue.

This study has limitations. We occasionally found it difficult to separate motivations for pursuing research careers from motivations for pursuing clinical careers, since fascination with a particular organ system or health issue was often at the root of both. We also did not analyze experiences by specific subpopulations such as race, ethnicity, academic rank, discipline, or country of origin.

Future work should explore these intersections longitudinally to refine and test the interventions we propose. By moving beyond recognition of the problem to specific institutional strategies, universities and funding agencies can build a more inclusive and sustainable research workforce. At a time when equity-focused initiatives face mounting challenges, adopting structured mentorship programs, bridge funding, transparent career-navigation supports, and family-responsive policies is both urgent and feasible.
