# Motivations to pursue careers in health research: A qualitative study of underrepresented faculty in the health sciences – CORRIGENDUM

**DOI:** 10.1017/cts.2026.10690

**Published:** 2026-02-06

**Authors:** Nancy Gauvin, Holly Thomas, Hemika Vempalli, Gretchen E. White, Natalia E. Morone, Audrey J. Murrell, Megan E. Hamm, Doris Rubio, Marie K. Norman

This notice is to advise that there is an error present in the author order in this original article. Marie K. Norman should be the last author in the order, as shown above.
